# Simultaneous alignment of short reads against multiple genomes

**DOI:** 10.1186/gb-2009-10-9-r98

**Published:** 2009-09-17

**Authors:** Korbinian Schneeberger, Jörg Hagmann, Stephan Ossowski, Norman Warthmann, Sandra Gesing, Oliver Kohlbacher, Detlef Weigel

**Affiliations:** 1 Department of Molecular Biology, Max Planck Institute for Developmental Biology, Spemannstrasse 37-39, D-72076 Tübingen, Germany; 2 Center for Bioinformatics Tübingen (ZBIT), Eberhard Karls University Tübingen, Sand 14, 72076 Tübingen, Germany

## Abstract

Genome resequencing with short reads generally relies on alignments against a single reference. GenomeMapper supports simultaneous mapping of short reads against multiple genomes by integrating related genomes (*e.g*., individuals of the same species) into a single graph structure. It constitutes the first approach for handling multiple references and introduces representations for alignments against complex structures. Demonstrated benefits include access to polymorphisms that cannot be identified by alignments against the reference alone. Download GenomeMapper at .

## Abstract 2

New software for the alignment of short-read sequence data to multiple genomes allows identification of polymorphisms that cannot be identified by alignment to a single reference genome.

## Rationale

Within the last few years, a variety of second- (or next-) generation sequencing technologies have been developed to enable analyses of small to medium-sized genomes within weeks or even days. The methods are now overcoming the disadvantages of short read length (currently the longest reads are obtained with the Titanium system produced by Roche/454 Life Sciences (Brandford, CT, USA) with Q20 at 400 bp) and a lower quality of individual reads with a dramatic increase in the total amount of data generated.

The initial resequencing of *Caenorhabditis elegans *and *Arabidopsis thaliana *(Arabidopsis) strains with Illumina reads [1,2] was recently complemented by genome sequences of several human individuals, generated with data derived from technologies from Illumina (San Diego, CA, USA), Applied Biosystems (Foster City, CA, USA), and Helicos (Cambridge, MA, USA) [3-10]. Even partial *de novo *assemblies of targeted regions within large genomes have been attempted [2]. However, short-read analysis of complex genomes is greatly aided by using a sequence backbone against which the short reads are aligned to find their genomic origin.

Different approaches for fast mapping of short reads have been suggested, including methods for indexing substrings of either the short reads or the reference sequence with the use of *k*-mers or spaced seeds (academic tools such as Bowtie, BWA, CloudBurst, MAQ, MOM, MosaikAligner, mrFAST, mrsFAST, Pash, PASS, PatMaN, RazorS, RMAP, SeqMap, SHRiMP, SliderII, SOAP, SOAP2, ssaha2 [2,11-28], and commercial tools such as ZOOM [29]). It has been reported that the current high demand for rapid alignments, to accommodate the flood of data generated by efforts such as the 1000 Genomes Project, can be met with new indexing strategies [16]. However, this is normally at the cost of not allowing complex alignments, including gaps.

For natural inbred strains of Arabidopsis, the high level of individual differences constitutes a substantial challenge. It has been estimated that several percent of the reference genome are either missing or very divergent in other strains of this species, which features homozygous genomes that are 25 times smaller than a haploid human genome [30,31]. This results in regions inaccessible to simple short-read alignments, in particular for alignment algorithms that do not accommodate many mismatches and gaps. New approaches supporting accurate alignments even in highly divergent regions are therefore sorely needed.

We note that the information derived from resequenced individual genomes is in itself useful for subsequent resequencing efforts, especially when the latter are at lower sequence coverage than the earlier efforts. Incorporating known polymorphisms increases the genome space against which the sample reads are aligned, which should greatly improve the mapping results. For example, an alignment suggesting a string of deleted bases in the focal genome becomes much more reliable if this deletion is known to exist in the population. The incorporation of such missing or inserted bases in the target/reference sequence not only would decrease the complexity of the alignments, but also would reduce sequencing costs, as more reads can be placed on the genome.

Apart from these practical reasons, aligning against only a single reference biases the analysis toward a comparison within the sequence space highly conserved with the reference. Taking into account all known genome variants would reduce this bias. Aligning reads against multiple genomes separately increases computation time and storage space and introduces new problems of merging and interpreting redundant results.

Here we present a new short-read alignment algorithm, GenomeMapper, which performs simultaneous alignments of short reads against multiple genomes. GenomeMapper assures high alignment quality, while competing in runtime with other short-read alignment tools. This is achieved by representing multiple genomes with a novel hash-based graph data structure against which the reads are aligned. To our knowledge, this constitutes the first approach for aligning a sequence against a graph of sequences rather than aligning two linear sequences. We also propose the first standards to tackle the problems arising from multiple references. GenomeMapper is currently the tool of choice for the Arabidopsis 1001 Genomes Project [32,33], and the default alignment option of the short-read analysis pipeline SHORE [2]. GenomeMapper has been used to analyze sequence reads derived from bacterial, plant, invertebrate, and mammalian genomes. To demonstrate the impact of adopting multiple genomes as the short-read alignment target, we describe the construction of a multiple genome sequence graph based on published polymorphisms of Arabidopsis [2]. We present the alignment and consensus sequence analysis of the Est-1 strain by using this graph and compare the results with the conventional approach of aligning the same set of reads against a single reference. We discuss the implications of our work for the analysis of more-complex reference sequences.

## GenomeMapper's indexing and alignment strategy

### Multiple genomes in one index

One way to decrease runtime for the generation of sequence alignments is to build index structures of either the reads or the reference sequence. To allow simultaneous alignments against multiple genome sequences, all target sequences have to be combined into one data structure. GenomeMapper achieves this goal by building a joint index of all genomes that are alignment targets. This index will be persistently stored, and, once compiled, the index does not need to be rebuilt for future alignment tasks.

The index is a simple hash-based mapping of *k*-mers (sequence signatures of 5 to 13 bp) to their locations within the target sequences. Each *k*-mer present in target sequences is unambiguously converted into a single integer, applying a two-bit representation of the four DNA nucleotides. Each hash key points to one hash value consisting of a list of all genome locations of the *k*-mer. Although this rather simplistic hash-indexing approach has some disadvantages compared with more recently developed strategies (*e.g*., Burrows-Wheeler indexing [16]), the latter are usually geared toward ungapped alignments and are not easily extendable to nonlinear structures imposed by multiple genomes. Further, spaced-seed approaches, implemented in tools such as SHRiMP or ZOOM, can be more sensitive [34]. However, when these approaches are applied to real data, they do not result in a substantial increase in the number of alignments compared with an approach with contiguous seeds followed by a complex alignment, because contiguous seeds are usually chosen short enough (*i.e*., 9 to 12 bp) for anchoring and subsequent aligning of reads (see later for comparison with other mapping tools).

Mapping indices tend to require a large amount of random access memory (RAM). Current computer servers usually allow multiple processors to share physical RAM. To avoid the unnecessary overhead of loading the same index multiple times, GenomeMapper makes use of memory-mapped files, allowing computer processes to share the same index structure within the memory. This reduces the overall memory footprint when running several instances of GenomeMapper in parallel.

### Index graph creation

**Figure 1:**
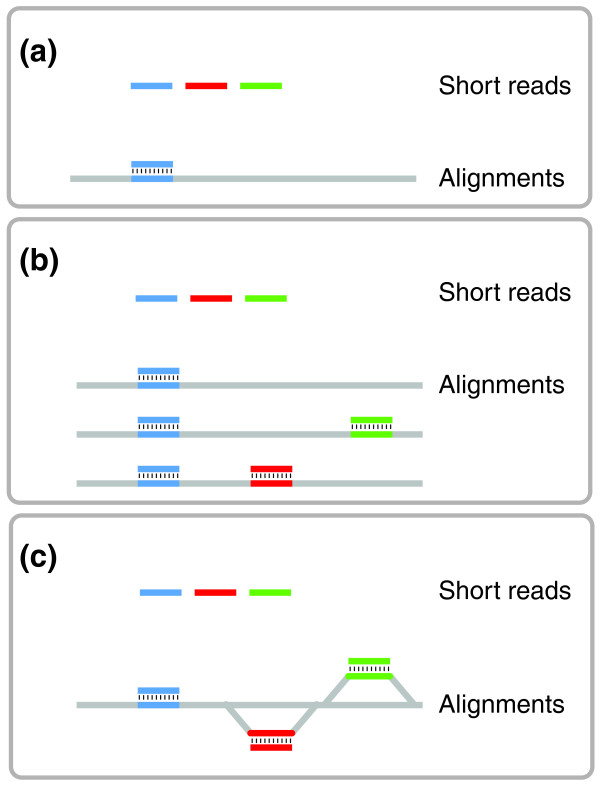
Efficient alignments against multiple genomes. **(a) **Only reads that are sufficiently similar can be aligned against a single reference. **(b) **Separate alignment against multiple genomes allows access to divergent regions, but results in redundant alignments of reads that match all targets (blue). **(c) **Alignments against a graph index representing multiple genomes provide access to divergent regions without redundant alignments.

The input for GenomeMapper's index-creation step consists of the sequence of one of the genomes and a list of differences in the other genomes compared with the first one (*i.e*., one FASTA file and a list of single-nucleotide polymorphisms (SNPs) and indels of every additional genome). Each position not explicitly annotated as different is assumed to be identical in all of the genomes, and will therefore be stored only once. This is important to avoid redundant alignments to several genomes. Divergent sequences are stored separately for each of the genomes. Identical regions, which are represented once, must be connected with polymorphic regions, which are represented by branches in the index. Hence, the reference loses its linear/sequential characteristic, but rather forms a sequence graph. Note that none of the genomes represents "the reference" (Figure 1).

**Figure 2:**
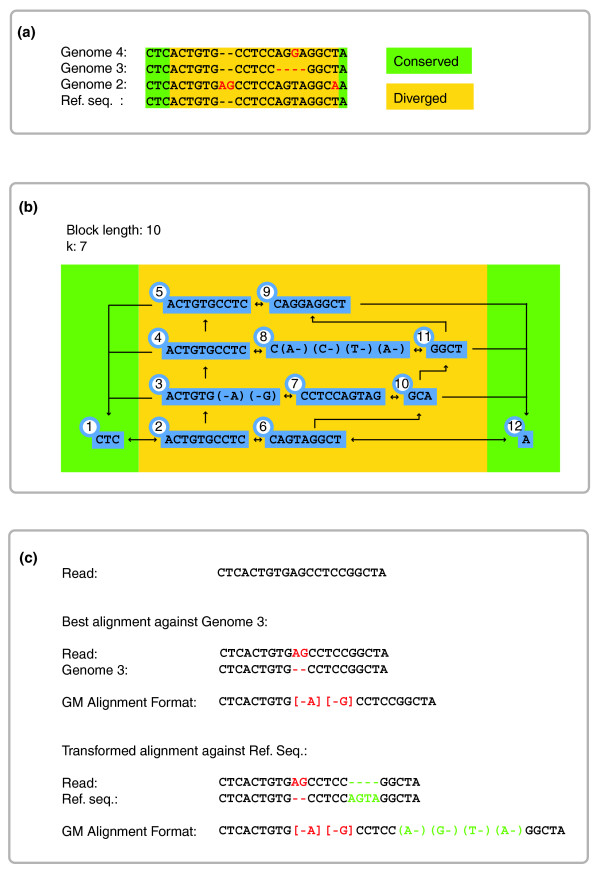
GenomeMapper's graph index structure. **(a) **Examples of orthologous sequences in four divergent genomes. Sequences at the beginning and end of each fragment are shared (underlaid with green boxes). Divergent regions start *k*-1 positions (in this case, six positions) before the first true variable position, to account for the *k*-mer length used for the hash-key calculation. **(b) **Graph structure created by these sequences, with *k*-mer length 7, and maximal block length of 10 (instead of 256) for reasons of illustration. The number attached to each block is its unique identifier. Note that blocks do not occupy their maximal block length after an indel, exemplified by blocks 3 and 8. Blocks 1 and 12 correspond to sequences identical in all four genomes and are present only once in the index structure. Arrows between the blocks visualize the edges between the nodes in the genome graph as they are stored in the block table [see Table S1 in Additional data file 1]. **(c) **Alignment of a read against the most similar genome, Genome 3, with a 2-bp insertion. Although the insertion also is observed in Genome 2, the 4-bp deletion downstream in Genome 3 makes the read more similar to it than to Genome 2. The transformed alignment of the read against the original reference sequence (Ref. seq.) includes the 4-bp deletion (as supported by Genome 3) given in parentheses (green), whereas the 2-bp insertion (which is supported neither by Genome 3 nor by the reference sequence) is annotated like a mismatch by using square brackets.

To store this information efficiently, each of the genomes is partitioned into non-overlapping sequence blocks of up to 256 bp, which represent the genomic sequence of all genomes. The connections of blocks to their neighbors allow continuous reconstruction of each genome. Invariant regions will be represented by one block only. Every variant, including all SNPs, will trigger the formation of branches, which constitute the parallel blocks that account for the nonlinearity of the genome graph (Figure 2a and 2b). Because complex differences such as inversions or duplications can always be defined as combinations of deletions and insertions, they can be readily incorporated into a graph index.

A unique identifier for each block allows a constant look-up time in a table that stores all relevant block information. In addition to referring to the genomes, of which it is a part, each block encodes for its sequence, the connections to its neighboring blocks, and the position within the genome. Each block thus harbors the genome sequence of all or a subset of genomes with identical sequences within the respective region. The block table is the implementation of a sequence graph, where the blocks represent the nodes, and the connections between them, the edges (Figure 2b). We refer to this table as *genome graph*. A comprehensive list of all features stored in the block table is given in Supplemental Table S1 [see Additional data file 1].

Generating different genome graphs with a different number of diverged genomes shows that the increase of a new sequence, and thus additional blocks, decreases with every new genome added; thus, the genome graph is less memory expensive than storing the genomes separately [see Additional data file 1].

Because all relevant information is stored in the genome graph, the positional information attached to each *k*-mer in the hash described earlier (linking each *k*-mer to its locations in the genome) must merely store the block identifier (represented by 3 bytes) and the position within the block (1 byte). Based on this information, the position of every base within each of the genomes can be inferred. The 4-byte encoding accommodates a combined length of all unique sequences of up to 4 Gb.

Efficient read mapping requires that each *k*-mer generated from one of the sequences in the genome graph can be queried for its locations in constant time. This is achieved by building a hash table connecting the *k*-mer (hash key) to its positional information in the genomes (hash value). Each hash key refers to a list of entries. Each of these entries stores a block identifier and a block position, allowing a unique positioning of each *k*-mer.

### Need for complex alignments

Earlier studies showed that, in a random comparison of two natural Arabidopsis strains, typically one SNP occurs for every 200 bp. In addition, by using early-generation Illumina single reads, more than 60,000 small indels (1 to 3 bp) and 10,000 indels of up to several hundred base pairs have been detected in two strains, presenting a lower boundary for the degree of polymorphism in this species [2].

Mismatches in alignments result not only from sequence differences, but also from sequencing errors. The error probability of Illumina sequence reads has been shown to be less than 1% for most, but not all parts of the read [2]. In comparison with the rate of natural variation in Arabidopsis, mismatches from errors in individual reads outnumber true SNPs approximately 17 to 1, whereas true gaps are almost as frequent as gaps resulting from sequencing errors [see Additional data file 1].

To avoid misplacement of individual reads, some mapping tools favor alignments in which the cumulative base quality of mismatching bases is low [21]. With respect to the high level of natural differences in Arabidopsis, such a strategy could bias alignments away from polymorphic regions. GenomeMapper instead performs, for each read, an alignment based on dynamic programming similar to the Needleman-Wunsch alignment algorithm (see [35] and Additional data file 1 for modifications). Our method ensures that all alignments within a given number of mismatches and gaps are reported, provided that they share at least one identical substring of length *k *when using a *k*-mer index. No other constraints are imposed on the number of mismatches, gaps, or base call quality. By default, GenomeMapper aligns against all instances of a repeat, but it also can be instructed to align only against a subset of them.

In our experience, resequencing projects of bacterial or medium-sized eukaryotic genomes such as those of Arabidopsis strains do not benefit from using alignments other than the optimal ones. Nonetheless, GenomeMapper can be configured to report not only the best scoring alignments, but also all hits within the specified range of mismatches and gaps (all-hits instead of best-hits strategy). As expected, this comes with an increase in runtime, especially for highly repetitive genomes.

### Aligning sequences against the graph

GenomeMapper's alignment procedure is partitioned into three steps, including speed optimization. The optimization bypasses the costly calculation of alignment matrices without a decrease in sensitivity and is based on two observations: first, a dynamic programming alignment is required only if the best alignment involves gaps; and, second, the frequency of gaps is lower than that of mismatches. This is the case both for sequencing errors in Illumina reads and for true polymorphisms. To cope with this, GenomeMapper applies a higher penalty for gaps than for mismatches. Therefore, alignments with a penalty lower than the gap penalty do not require dynamic programming. The optimization cannot be applied in an all-hits strategy including gapped alignments and will not increase speed if the best alignment features gaps.

In the first step of the alignment procedure, GenomeMapper scans the hash index for *k*-mers identical between read and genome graph to detect quickly all genomes and locations with nearly identical alignments. In the second step, GenomeMapper determines the location and sequence of *nearly identical maximal substrings *(NIMS) between read and genome graph. GenomeMapper will finally perform a *k*-banded alignment by applying dynamic programming to ensure a consistent gap placement [see Additional data file 1].

In detail, GenomeMapper starts by calculating the hash keys of a predefined set of non-overlapping *k*-mers of the read sequence and retrieves their genomic positions from the hash index. The pair, consisting of a *k*-mer along with one of its positions in the genome, is referred to as *hit*. If the best alignment of a read contains up to one mismatch less than the number of non-overlapping k-mers fitting into the read, at least one hit within this alignment can be computed (see [36] and Additional data file 1). Each hit serves as the seed for an ungapped alignment comparing the unmatched parts of the read with the target sequence.

If the first step does not reveal a valid alignment, which is always optimal because of the prerequisite that one mismatch is less penalized than one gap, GenomeMapper starts calculating hits not only for a subset, but also for each of the *k*-mers within the read sequence. If two hits are adjacent in the read and in the genome graph, they will be merged, resulting in so-called *extended hits*. If a single mismatch between read and genome sequence is adjacent to extended hits on either side, GenomeMapper can bridge this mismatch by merging the extended hits now harboring this mismatch. Once all hits are maximally extended (they now constitute NIMSs), the read has to be aligned against the regions determined by each of the NIMS, aborting as soon as the best possible alignment will be worse than the mismatch and gap constraints [see Additional data file 1].

To retrieve the genomic sequence for the alignments, GenomeMapper must follow the links between blocks. Starting from the block harboring the hit or NIMS, GenomeMapper follows the edges of the genome graph to generate a target sequence for the alignment. If multiple blocks reside next to one of these blocks, each of the branches will generate a separate target sequence for an independent alignment. Note that GenomeMapper will not concatenate sequences from different genomes. The alignment phase is implemented with an efficient parallelization, which substantially reduces runtime. It is distributed in a master-slave model on a shared-memory architecture. All alignment threads can access the genome data and the read data. The master thread distributes individual hits by signaling each alignment thread and collects the results. The number of threads used by the parallel implementation is a user-defined parameter that can be adjusted to the hardware.

The parallel version of GenomeMapper relies on POSIX threads to manage the individual compute threads efficiently. POSIX threads are available for all relevant platforms (including Linux, Mac OS, and Windows).

### Representation of the alignments

Independent of the algorithm used to detect the best alignments, GenomeMapper will report two different representations of the alignment. The first one constitutes the alignment of the read against the genome to which it is most similar (reference-free alignment). Because commonly used tools for alignment consensus analysis such as MAQ, Mosaik, SHORE, and VAAL [1,2,18,37] report base calls based on the location relative to one reference sequence, GenomeMapper implements a second alignment representation, which transforms the strain alignment into an alignment against the reference sequence. This reference-based alignment can then be used as input for one of the tools mentioned earlier. Which of the genomes constitutes the 'reference sequence' is defined in the index creation. As the reference sequence is not necessarily the most similar sequence to the read, the reference-based alignment can feature more mismatches and gaps than the strain alignment and can exceed the user-defined constraints.

This transformation generates two categories of mismatches in the reference-based alignment. The first category contains mismatches that are unique to the read sequence. The second consists of mismatches identical between the read and the strain it was aligned with, but different from the reference sequence. Such mismatches are more likely to represent true polymorphisms, because they have already been previously observed. GenomeMapper indicates the different types of mismatches by using round and square brackets (Figure 2c). We have updated SHORE's [2] consensus analysis to exploit this additional information (see section, *Impact on Resequencing*).

### Position descriptors for reference-free and reference-based alignments

An alignment is typically anchored by the position of the 5' nucleotide in the target sequence at which the alignment starts. Because different genomes may feature indels of different lengths, however, even for identical sites, positional information can become ambiguous. The decision for one of the locations only (*e.g*., that of the reference genome) would overvalue the reference.

Currently the sole community-wide accepted description of a genomic location is the corresponding nucleotide within the reference sequence, which easily accommodates gaps, but not insertions, relative to the reference. We therefore implemented two position descriptors into GenomeMapper. The first refers to the particular genome against which the alignment was performed (the strain alignment). The second represents the position of the alignment against the reference (the reference alignment). Insertions are annotated by using the upstream reference position followed by the position of the inserted nucleotide within the insertion, separated by a decimal point (*e.g*., "80359.12" describes the 12th nucleotide within the insertion after position 80359 of the reference). Strain alignments transformed to reference alignments lose their reference-free characteristic and therefore are immediately comparable with conventional mapping results.

### Comparison with other mapping tools

**Figure 3:**
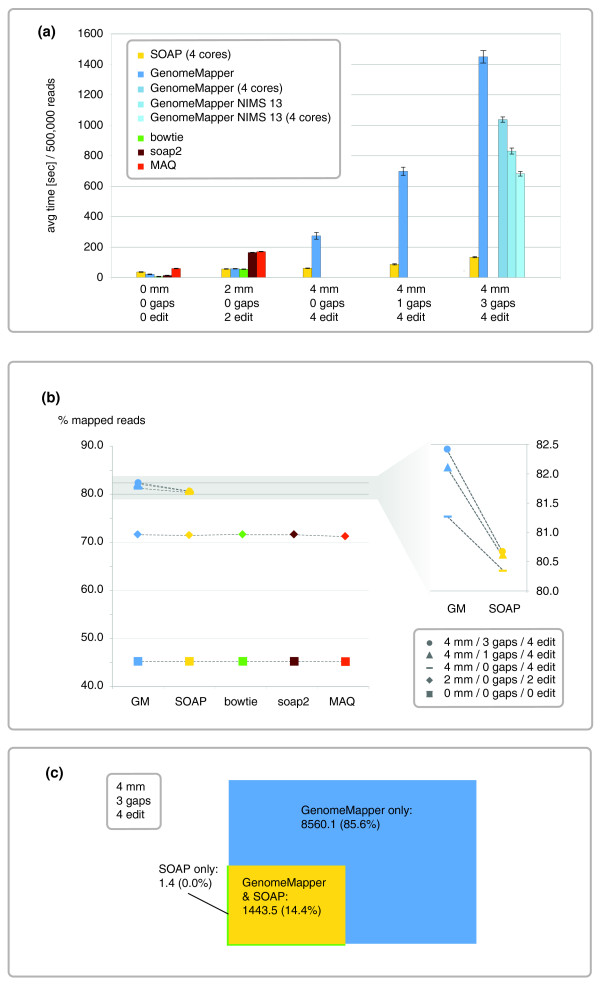
Performance of GenomeMapper compared with that of other short-read alignment tools. **(a) **Runtime, measured as wall clock time between invocation and termination of the program, averaged from 10 independent tests with different random sets of 500,000 short reads from Est-1. The worst test was excluded from average calculations. Error bars indicate standard deviation. *mm*, *gaps*, and *edit *refer to the maximal number of mismatches, gaps and edit operations allowed. GenomeMapper was run with four different parameter settings: the serial version; the parallel version on four cores; the serial version merely aligning NIMS of length 13 or longer; and the parallel version aligning only NIMS of length 13 or longer. SOAP was found running on up to four CPUs instead of only one CPU, as configured with the command line (option -p). **(b) **Average sensitivity, measured as the percentage of aligned reads. Only GenomeMapper and SOAP can perform gapped alignments. **(c) **Average sensitivity of alignments, allowing three gaps and four mismatches with a combined maximum of four edit operations, measured as number of reads with gapped alignments. Fractions refer to the number of all reads with gapped alignments.

GenomeMapper also can be used for alignments against a single target genome. This allowed us to compare runtime and sensitivity of GenomeMapper (version 0.3.1s) with those of four other popular mapping tools: SOAP (version 1.11 [19]), soap2 (version 2.01 [20]), bowtie (version 0.9.8 [16]), and MAQ (version 0.7.1 [18]). SOAP and MAQ were previously compared with bowtie [16], but with a human target. Here we aligned against the Arabidopsis Col-0 reference genome [38] with seed length set to 12. All tests were performed on 10 independent read sets, each consisting of 500,000 reads randomly sampled from reads generated in this work for the Arabidopsis Est-1 strain (see later). We tried to run all alignment tools with optimal parameters to achieve the best possible sensitivity and runtime [see Supplemental Table S2 in Additional data file 1 for command lines of all runs]. To make them directly comparable with GenomeMapper, we set SOAP, soap2, and MAQ to report all repetitive best hits rather than a random subset of them, even though this comes with an additional investment in runtime. All tests were performed on a compute server with eight cores (two AMD Opteron quad core processors) and 32 GB RAM. Figure 3 compares average runtimes, measured as the wall clock, as well as sensitivity of all alignments and of gapped alignments, both measured as the number of reads that could be aligned. As this analysis is based on real data for which no gold-standard sequence information is available, nothing is known about the true origin of the DNA reads. We therefore took the fraction of aligned reads as a proxy for sensitivity.

Without allowing any mismatches, little difference in runtime or in sensitivity was found between the alignment tools, with GenomeMapper being slower than bowtie and soap2, but faster than SOAP and MAQ. Allowing two mismatches caused similar increases in runtime for all tools. With respect to sensitivity, more than 99% of the differences in the reads that could be aligned with up to two mismatches resulted from different strategies in aligning ambiguous base calls (Ns). SOAP, for example, aligns Ns without an alignment penalty.

Different from SOAP, GenomeMapper's runtime was drastically affected by allowing additional gaps (which are not accommodated by the other tools tested) (Figure 3a). The first reason for this disparity is the different alignment strategy. SOAP allows neither gaps combined with mismatches nor multiple gaps in the same alignment, whereas the dynamic programming alignment in GenomeMapper supports any combination of gaps and mismatches. Second, even though SOAP was set to run on one processor (option -p was set to 1), we found it running in parallel on up to four CPUs, and therefore using more computational power than the other tools.

By applying GenomeMapper's parallelization set to run on four cores, runtime was reduced significantly. Parallelization is geared toward complex alignments and did not reduce runtime for ungapped alignments. Another way to reduce runtime is offered by skipping alignments triggered by NIMS/hits of length 12 (seeds that could not be extended by at least one base, option -l, indicated by "NIMS 13" in Figure 3a), but this came at a cost of sensitivity being reduced by 0.6%.

Compared with SOAP, GenomeMapper's more accurate alignment method resulted in higher sensitivity (Figure 3b; compare results for 4 mm/1 gap and 4 mm/3 gaps). Considering only gapped alignments, GenomeMapper aligned more than 5 times as many reads as SOAP (Figure 3c), whereas only one of 500,000 reads was aligned by SOAP, but not by GenomeMapper. This difference showcases GenomeMapper's ability to combine multiple gaps with mismatches in the same alignment.

Note that the reads used for benchmarking had been quality trimmed. This removes the common trend of read endings having increased chances of harboring mismatches because of higher error rates. Untrimmed reads with additional mismatches would have almost completely prohibited SOAP from performing gapped alignments. This is expected to be even more an issue with longer reads.

GenomeMapper's relatively high runtime when allowing a large number of gaps and mismatches is explained mostly by the enormous number of alignments performed once optimizations could not reveal the best alignment. Nonetheless, accurate alignments are important for correct read placement in regions of high divergence and therefore justify the performance loss. Whereas aligning against a genome graph comes with additional computational costs, it greatly increases sensitivity. One can compensate for increased runtime with computing power, but reads that are never correctly aligned in the first place are lost for further analyses.

### Impact on resequencing

To examine the practical relevance of graph-based alignments against multiple genomes, we compared performance with a conventional single-reference approach by using reads from the genome of Arabidopsis strain Estland-1 (Est-1) from Estonia, generated in the *Arabidopsis thaliana *1001 Genomes Project [see Additional data file 1]. The 47.7 million alignable single-end high-quality reads were produced on an Illumina Genome Analyzer. After quality trimming of the reads to 36 to 42 bp, the average depth of genome coverage was 13 fold.

**Figure 4:**
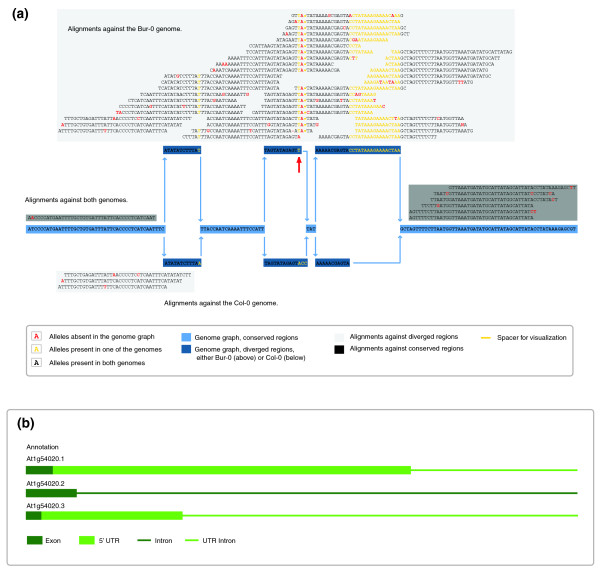
Alignments against a 17-bp insertion present in a nonreference genome. **(a) **Alignments of Est-1 reads against the graph of Arabidopsis chromosome 1, reference positions 20,166,584 to 20,166,747. Alignments against both the Col-0 reference and the Bur-0 variant genomes are highlighted in dark gray; alignments of reads aligning best against a single genome are highlighted in light gray. Most reads align against the Bur-0 allele, suggesting that Est-1 is more similar to Bur-0 at this locus. In particular, the 17-bp insertion found in Bur-0 is supported by the Est-1 reads. Because of the alignment constraints (maximum of four edit operations), these alignments could not have been performed against the Col-0 sequence only. Within the second divergent region, indicated by a red arrow, Bur-0 has a complex change, ACC->T, relative to Col-0, with Est-1 featuring a third allele, ACC->TA. Because this change is near the 17-bp insertion, only a subset of the alignments would have been found with single reference alignments only. For simplicity, Tsu-1, which also is included in the graph target, is not shown here. **(b) **Annotation of this region with respect to the Col-0 reference genome.

We first used the reference Arabidopsis Col-0 sequence (TAIR8 [38]) as the alignment target. In the second analysis, we included two Arabidopsis genomes, Bur-0 and Tsu-1 (see Figure 4). Previous Illumina single-read sequencing and comparison against the Col-0 reference had revealed 570,100 and 502,036 SNPs, as well as 48,999 and 47,765 indels of up to 3 bp, respectively [2]. In addition, 16,463 and 3,007 longer indels of up to 641 bp had been discovered from targeted *de novo *assembly of highly polymorphic regions [2]. These two genomes differ from the reference by 0.5 to 0.6%, which reflects a lower bound of sequence divergence, given the limitations of short-read analyses.

The Bur-0 and Tsu-1 genomes, together with the Col-0 reference genome, were used to build a multiple genome graph. To take advantage of the additional information produced by the graph-based alignments, and to make it comparable to a single reference analysis, we updated SHORE [2], our genome-resequencing analysis pipeline [see Additional data file 1]. This included incorporation of GenomeMapper's transformed alignment representation, different scoring schemes for previously known and newly discovered polymorphisms, and the support of indels up to any length, restricted only by the maximal indel length within the known genome space.

More than 1% of all reads, 0.51 million reads, could be aligned to the genome graph, but not to the single reference. These additional alignments resemble highly divergent regions of Est-1, which are particularly interesting, but also constitute the regions that are least accessible to conventional methods. Compared with the "reference only" alignments, the graph alignments increased the number of recovered SNPs by 15%, of deletions by 22.6%, and of insertions by 37.2% (Table 1). In particular, 1,551 deletions and 1,841 insertions longer than 3 bp, with a maximum length of 641 bp and 281 bp, known from previous *de novo *assembly of larger indels in Bur-0 and Tsu-1 [2], were detected. Only a small subset of the long indels was represented in the "reference only" analysis (two 3-bp deletions can modify the sequence in the same way as one 6-bp deletion). Because of the limitation of three gapped positions per alignment, the vast majority of long indels could not be discovered with the conventional "reference only" alignment. These observations illustrate that indel detection is not limited by alignment constraints, but only by the data included in the genome graph.

**Table 1:** Recovery of Est-1 variants by using SHORE

		**Predicted by both analyses^a^**	**Private to genome graph analysis**	**Private to reference-only analysis**	**Total gain in genome graph analysis**
SNPs		401,158	66,264	5,423	15.0%

Deletions	All	25,926	6,807	778	22.6%
	1-3 bp	25,865	5,256	778	16.8%
	≥ 4 bp	61	1,551	0	2,542%

Insertions	All	22,305	9,220	678	37.2%
	1-3 bp	22,285	7,379	678	29.2%
	≥ 4 bp	20	1,841	0	9,205%

^a^Includes variants predicted by graph analysis that have been found in the single-reference analysis in the same sequence context, although with a differing position, resulting from ambiguous alignments. Some of the variants longer than 3 bp could be reassembled in the single-reference analysis, by combining shorter indels.

The reliability of variant detection was improved as well, with 244,101 SNP calls made in the "reference only" analysis having additional support from one of the additional genomes in the graph (11,382 and 16,958 for deletions and insertions, respectively). Similarly, recall rates for 1 to 3 bp indels were drastically increased.

Validation results for single-reference and genome-graph analysis based on 600 kb of dideoxy sequences distributed throughout the Est-1 genome [39] are shown in Table 2. In a typical Arabidopsis strain, about 85% of SNPs are accessible to analysis with 36-bp single-end short reads, with the remainder being located in repetitive regions [2]. Of 2,316 SNPs in the validation set, 85.2% were called by using genome-graph analysis, an increase of more than 7% compared with the single-reference analysis at a similar error rate of less than 0.5%. Recall rates for indels were increased even more, by 14.8% for insertions and 8.4% for deletions.

**Table 2:** Validation of polymorphism predictions in Est-1

			**Graph analysis**		**Single-reference analysis**	
				
		***N* ^a^**	**Recall^b^**	**FDR^c^**	**Recall^b^**	**FDR^†^**
SNPs		2,316	85.2%	0.4%	77.5%	0.4%

Deletions	All	183	53.6%	2.0%	38.8%	2.7%
	1-3 bp	132	68.2%	2.2%	53.8%	2.7%
	≥ 4 bp	51	15.7%	0.0	0	n/a

Insertions	All	167	53.9%	2.2%	45.5%	1.3%
	1-3 bp	128	66.4%	2.3%	59.4%	1.3%
	≥ 4 bp	39	12.8%	0.0	0	n/a

^a^Number of known variants in 600 kb of dideoxy sequence data from [38]. ^b^Ratio of confirmed to the sum of confirmed and missed predictions of the respective kind; indicates sensitivity of method. ^c^False discovery rate, percentage of erroneous calls.

For a final comparison, we aligned all Est-1 reads against the three known genomes separately, with the Bur-0 and Tsu-1 genome sequences generated by introducing all known variations into the reference Col-0 genome. As expected, nearly the same set of reads could be aligned, but the graph alignments were 21.3% faster than the serial alignments. This improvement would be even greater if one took into account the additional analyses needed for merging and filtering of separate and redundant alignments.

The results of the graph analysis of Est-1 can be downloaded from the 1001 Genomes portal [33] and from TAIR [40].

## Discussion

The first goal for short-read mapping tools was the design of efficient alignment algorithms that were faster than the speed with which raw data were produced. Considering that intraspecific sequence differences are often more substantial than previously anticipated, a major challenge is the requirement not to disregard or misplace too many reads. With the rapidly increasing knowledge of variants, one could simply align against all known genomes for a species separately. This would not require any new methods, but it comes with the overhead of redundant alignments in conserved regions. We have shown that graph alignments are already superior with information from only two divergent genomes added to the first genome sequence produced for Arabidopsis. This advantage should become much more drastic once hundreds of genomes are incorporated into the graph structure. In addition, this should improve the workflow, as the separate handling of hundreds of separate references would become increasingly impractical.

We demonstrated that short-read alignment against a complex graph representing multiple genomes not only is possible and produces meaningful results, but also provides access to regions that are highly divergent from the first reference. In addition, our approach reduces the number of false-positive SNP calls caused by misalignments near indels [2]. To our knowledge, this constitutes the first approach that efficiently incorporates multiple references and solves resultant problems. We note in addition that the representation of multiple genomes in a complex graph structure is not restricted to short-read mapping or intraspecific analyses. Other applications are easily conceivable (*e.g*., accurate local and global alignments of longer reads (up to whole genomes) against all known genomes of a species or even against a structure representing groups of related species), enabling analysis of metagenomic samples in one step. Likewise, read alignments against splice graphs representing known isoforms with differing exon-intron junctions would be beneficial for mRNA analysis.

Once the species-wide genome graph of Arabidopsis covers most common variants (see the *Arabidopsis thaliana *1001 Genomes Project [32,33]), resequencing of newly collected material will become easier, as fewer inaccessible regions remain. A prerequisite for this are universal and community-wide accepted positional descriptors of insertions, for which we have advanced a proposal in this work.

### Ongoing development

The steady increase in read length will improve the likelihood that a given read spans a region of complex differences relative to the first reference. Although no theoretic limitation exists for the lengths of global alignments (GenomeMapper currently allows reads of up to 1,000 bp with unlimited numbers of mismatches or gaps), allowing more and more mismatches and gaps would strongly affect runtime. This could be addressed by further increasing the efficiency of the parallelization, which is already tuned to reduce runtime for long-read alignments with numerous gaps and mismatches.

Another challenge that is conceptually similar to matching known SNPs relative to the reference emerges from bisulfite treatment of DNA samples for methylome analysis [41]. The presence of cytosines that have been converted to thymines by bisulfite can be implemented as mismatches without penalty. This is currently being incorporated into GenomeMapper and will be supported in future versions.

## Abbreviations

Bp: base pair; GB: gigabytes; indels: insertions and/or deletions; k-mer: sequence signature; NIMS: nearly identical maximal substrings; POSIX: Portable Operating System Interface for Unix; RAM: random-access memory; SNP: single-nucleotide polymorphism; TAIR: The Arabidopsis Information Resource.

## Authors' contributions

KS and DW designed the study. KS and JH developed and implemented GenomeMapper. SO suggested optimizations resulting in major speed improvements, extended SHORE for the analysis of genome-graph alignments, and performed the Est-1 analysis together with JH and KS. SG implemented the parallelization, as discussed with OK. NW did the plant work and generated the Illumina sequencing library. KS wrote the manuscript with help from all authors.

## Additional data files

The following additional data are available with the online version of this article. Additional data file 1 describes supplementary methods and discussions, as well as tables listing the features of genome graph structure and the command lines used for comparison of the different alignment programs.

## Supplementary Material

Additional data file 1 Supplementary methods and discussions, as well as tables listing the features of genome graph structure and the command lines used for comparison of the different alignment programs. Click here for file
