# Supplementary material for: Simultaneous alignment of short reads against multiple genomes
Source: Genome Biol. 2009 Sep 17;10(9):R98. doi: 10.1186/gb-2009-10-9-r98 (PMC2768987; doi:10.1186/gb-2009-10-9-r98)
Supplement: Additional data file 1 — Supplementary methods and discussions, as well as tables listing the features of genome graph structure and the command lines used for comparison of the different alignment programs. [file gb-2009-10-9-r98-S1.DOC]

# Simultaneous alignment of short reads against multiple genomes

Schneeberger K, Hagmann J, Ossowski S, et al.

# Supplemental Methods

## Biological material, library preparation, and SBS sequencing

Seed stocks for Est-1 were from sibling plants of those used for the resequencing array study (Arabidopsis Biological Resource Center stock numbers CS22683) (Clark et al. 2007). Leaves (1.0 gram fresh weight) were ground in liquid Nitrogen with a mortar and pestle, and DNA was purified with the DNeasy Plant Maxi Kit (cat. no. 68163; Qiagen Inc., Valencia, CA) according to the manufacturer’s instructions eluting with 750 µl of buffer AE. The DNA solution was concentrated to 100 ng/µl using a Speedvac.

From 5 µg of the resulting DNA (~50 µl), single-end SBS libraries were prepared with the Genomic DNA Sample Prep Kit (cat. no. FC-102-1001; Illumina Inc., San Diego, CA) following the manufacturer’s instructions (Part # 1003806 Rev. A). Briefly, DNA was sheared by nebulization for 6 minutes with N2 gas at a pressure of 32 psi; then the ends of the sheared fragments were repaired, an A residue to the 3’ end of the blunted fragments was added and the supplied adaptors were ligated. The reaction was resolved on a 2% agarose gel (including 400 ng/ml ethidium bromide) run in TAE buffer for 60 minutes at 120 volts. Adapter-modified DNA fragments between ~150 to 200 bp in size were excised from the gel under illumination from a Dark Reader (Clare Chemical Research), the DNA was isolated with a Qiagen Gel Extraction Kit (Qiagen, Hilden, Germany) and subjected to 14 cycles of enrichment PCR using the supplied primers 1.1 and 1.2. After quantification with a NanoDrop Spectrophotometer (Peqlab Biotechnologie GmbH, Germany), the DNA was then diluted to a 10 nM stock in EB buffer (Qiagen) supplemented with 0.1% Tween-20 and stored at -20˚C.

## Cluster preparation on flowcells and SBS sequencing

Cluster generation was performed on a Cluster Station (cat no. SY-301-2001; Illumina) with the recipe “One_step_full_protocol_v3.0” and reagents from Cluster Generation Kits (cat. no. FC-103-1002; Illumina) according to the manufacturer’s instructions.

In total, we used 7 lanes: 3 were loaded with 4 µl and 4 with 6 µl of the 10 nM working stock and cluster densities between 29,100 and 41,300 clusters were obtained.

Sequencing was performed in March 2008 on a Genome Analyzer (cat. no. SY-301-1001; Illumina) using the then current 36 Cycle Solexa Sequencing Kit according to the manufacturer’s instructions (SCS1.01, Scanbuffer v2); the respective *_AF4.xml file was modified to allow extension and imaging of 43 cycles.

## The decrease in the increase of new sequence

In order to show that storing genomes separately is more memory consuming than building a genome graph we generated genome graphs incorporating the information of one, two, or three strains in addition to the reference. A graph featuring only the reference genome has 100% of conserved sequence. Incorporating a single additional strain reduced the conserved genome space by about 5%, namely to 94.3% (for Bur-0) or 95.1% (for Tsu-1). Simultaneously adding both Bur-0 and Tsu-1 resulted in a further decrease of conserved genome space, but by less than 4%, to 91.6%. Adding a third genome, Est-1, reduced conserved genome space by less than 2%, to 89.9%. The reason behind this is that variants are in linkage disequilibrium and not randomly distributed among individuals.

## Number of mismatches originating from real genetic variants vs. sequencing errors

Based on the single reference alignments of the Est-1 reads, we separated all mismatches (and gaps) at positions resulting in SNP calls (or indel calls) from the mismatches (and gaps) at positions with a high confidence reference call. The outcome of this analysis depends on the divergence between sample and reference sequence, as well as on the quality of the sequencing run. It does not allow for general conclusion about the Illumina GA sequencing technology.

## Selection of *k*-mers for identification of highly identical alignments

As a first step in the alignment procedure and in order to avoid calculation of the computationally expensive alignment matrix for alignments with a very low number of mismatches, GenomeMapper calculates hits from a subset of the *k*-mers of reads. The selected *k*-mers are non-overlapping *k*-mers at the read positions 1, *k*+1, 2*k*+1… The last *k*-mer will be calculated starting at read length-*k* positions. Each hit will then become target of a simple alignment algorithm comparing the read and genome sequence surrounding the hit, not allowing for any gaps. The rationale behind this approach is to identify all alignments, which feature one mismatch less than non-overlapping *k*-mers fit into the actual read (compare to *q*-gram lemma [35]).

Since this does not consider gapped alignments, it can result in alignments featuring worse scores than alignments with gaps. Therefore the more accurate *k*-banded dynamic programming alignment algorithm has to be applied if no alignments with less than 2 mismatches were detected (or with one mismatch less than non-overlapping *k*-mers fit into the read if no gaps are allowed).

## Needleman-Wunsch-like *k*-banded alignment

GenomeMapper employs *k*-banded dynamic programming alignments. The alignment constraints of the upper limit of gaps implicates that all valid traceback paths will not involve alignment matrix cells which are more than *k* vertical or horizontal trajectories distant from the main diagonal. Thus, only *k* cells on either side of the diagonal have to be computed shaping a band with *k* being the number of allowed gaps.

GenomeMapper's alignment method is similar to the Needleman-Wunsch algorithm [34]. The only differences are modified traceback rules and the introduction of an abortion criterion. Since reads are aligned not only against the genomic region determined by NIMSs, but against an enlarged genomic region in order to allow for gaps, the start and end of the traceback routine is limited to the cells in the last and first row of the *k*-band and not only to the bottom-right and top-left matrix cell, respectively, as required by the Needleman-Wunsch algorithm.

If the number of allowed edit operations is exceeded before the alignment is finished, GenomeMapper will stop the computation. As the alignment matrix is filled column-wise and penalties are non-negative, its computation is aborted as soon as the best score of all cells in each column is worse than the score resulting from the maximal number of allowed gaps and mismatches.

Furthermore, if GenomeMapper runs in best-hit mode, the maximal number of edit operations is not only defined by the global adjustments as set from the command line, but are further restricted by the best alignment found so far. Every best alignment will update the restrictions for the upcoming alignments of the same read.

Every alignment meeting the constraints of mismatches, gaps and edit operations is stored and ranked by its score. The best-hit mode will only report the alignments with the highest score.

## Increased reliability of SNP calls

The reliability of SNPs increases drastically if prior knowledge about its existence is available. This prior knowledge is given through the incorporation of known SNPs into the alignment target and mismatches of reads against the reference supported by matches against the sequence of other genomes are annotated in the alignment in a different format than non-supported mismatches (e.g. a mismatch of A to T, non-supported: [AT], supported: (AT)). We reduced the minimum coverage requirement for SNPs and short deletions supported by other genomes from 3 to 1.

## SHORE implementing genome graph analysis

SHORE’s consensus algorithm works on a proprietary input format (called map.list) representing read ID, alignment and alignment locus, repetitiveness as well as read and alignment qualities. We have extended this format to store both the alignment of a read against the reference and against the best matching strain.

To tackle the problem of reads starting at positions not present in the reference, which therefore miss a proper start position, we extended the position information to represent inserted positions as the position upstream of the insertion followed by the position within the insertion with both numbers separated by a decimal point. For example, “20.12” describes the 12^th^ position in the insertion starting after position 20 of the reference.

The consensus prediction including reference-like, SNP and indel positions is performed identical to the decision tree algorithm used for single reference alignments described in an earlier work [2], with two exceptions: First, SNPs and deletions that are known from previously sequenced genomes have a decreased threshold for minimum coverage (1 instead of 3). Second, the limit for the length of deletions and insertions (equal to maximum allowed gaps) is removed completely. For insertions longer than the read length this means that covering reads can have no anchor in the reference sequence anymore but are completely contained within the insertion. In order to approximate the concordance of the consensus call for long insertions (reads supporting the insertion divided by all reads covering a position), we compare the maximum read coverage within an insertion to the total number of reads not supporting the insertion. This issue does not apply to deletions, because deletions are always spanned by a read and therefore concordance can be accurately calculated similar to reference or SNP calls.

‘SHORE consensus’ can be configured to run in graph mode with a single parameter. The output is identical to the ‘single reference’ analysis allowing for straightforward comparison of results.

# Tables

## Table S1

## Features stored in one block.

| **Feature** | **Size in bytes** | **Description** |
| --- | --- | --- |
| genome_pos | 4 | Start position of the block with respect to the sequence it is derived from. |
| ref_pos | 4 | Start position of the block with respect to the reference sequence. |
| Chr | 4 | Chromosome identifier |
| Strain | 4 | Genome identifier |
| indel_offset | 2 | Stores the position of the indel within the block |
| ins_pos | 4 | Stores the number of inserted bases upstream. |
| Seq | 256 | Genome sequence, stored as transformed alignment. |
| prev_block | 4 | Block identifier of preceding block |
| next_block | 4 | Block identifier of succeeding block |
| next_strain_front | 4 | Block identifier of the first block of the next diverged strain (in diverged regions only). This is the interconnection between the strains in Fig 2 and 4. |
| next_strain_end | 4 | Block identifier of the last block of the next strain (in diverged regions only) |

Each block is represented by one such entry in the block table. Since the block table is an ordered list, the blocks do not need to store their identifier, since it is encoded as their positions in the list (block table).

## Table S2

## Command lines used for the different alignment tools.

| GenomeMapper | serial: -E *mm* -M *mm* -G *gaps*  serial with NIMS length 13: -E *mm* -M *mm* -G *gaps* -l 13  4 cores: -E *mm* -M *mm* -G *gaps* -t 3  4 cores and NIMS length 13: -E *mm* -M *mm* -G *gaps* -l 13 -t 3 |
| --- | --- |
| SOAP | -s 12 -v *mm* -g *gaps* -w 10000 -c 0 -r 2 |
| Bowtie | Allowing for 0 mismatches: -v 0 --time --seed 8526367  Allowing for 2 mismatches: -v 2 --time --seed 8526361 |
| soap2 | Allowing for 0 mismatches: -M 0 -r 2  Allowing for 2 mismatches: -M 3 -r 2 |
| MAQ | Allowing for 0 mismatches: -n 1 –e 0 -C 513 -N  Allowing for 2 mismatches: -n 2 –e 80 -C 513 -N |

*mm* and *gaps* denote the maximal number of allowed mismatches and gaps, respectively. Not given, options for input, output and index files.
